# Who Deserves My Trust? Cue-Elicited Feedback Negativity Tracks Reputation Learning in Repeated Social Interactions

**DOI:** 10.3389/fnhum.2017.00307

**Published:** 2017-06-15

**Authors:** Diandian Li, Liang Meng, Qingguo Ma

**Affiliations:** ^1^School of Management, Zhejiang University Hangzhou, China; ^2^Beijing Xinsight Technology Co. Ltd. Beijing, China; ^3^Neuromanagement Lab, Zhejiang University Hangzhou, China; ^4^School of Business and Management, Shanghai International Studies University Shanghai, China; ^5^Laboratory of Applied Brain and Cognitive Sciences, Shanghai International Studies University Shanghai, China; ^6^Institute of Neural Management Sciences, Zhejiang University of Technology Hangzhou, China

**Keywords:** trustworthiness, trust game, social learning, event-related potential, feedback negativity

## Abstract

Trust and trustworthiness contribute to reciprocal behavior and social relationship development. To make better decisions, people need to evaluate others’ trustworthiness. They often assess this kind of reputation by learning through repeated social interactions. The present event-related potential (ERP) study explored the reputation learning process in a repeated trust game where subjects made multi-round decisions of investment to different partners. We found that subjects gradually learned to discriminate trustworthy partners from untrustworthy ones based on how often their partners reciprocated the investment, which was indicated by their own investment decisions. Besides, electrophysiological data showed that the faces of the untrustworthy partners induced larger feedback negativity (FN) amplitude than those of the trustworthy partners, but only in the late phase of the game. The ERP results corresponded with the behavioral pattern and revealed that the learned trustworthiness differentiation was coded by the cue-elicited FN component. Consistent with previous research, our findings suggest that the anterior cue-elicited FN reflects the reputation appraisal and tracks the reputation learning process in social interactions.

## Introduction

In many social interactions involving exchanges, trust and trustworthiness are essential components because social exchange relationship usually develops on trust where formal contracts are absent (Ashraf et al., 2006). Trust and trustworthiness foster reciprocity and pro-social behaviors and contribute to better economic outcomes on both individual and organizational levels (Charness et al., 2011; Johnson and Mislin, 2011).

In general, one trusts another because the latter is believed to be trustworthy. The strength of this belief is affected by various factors of the two parties. Studies have shown that one’s gender, race and socioeconomic status influence how much she/he trust others (Alesina and La Ferrara, 2002; Chaudhuri and Gangadharan, 2007). It is also found that the level of trust is different among different countries and regions (Willinger et al., 2003; Johnson and Mislin, 2011). One’s trustworthiness, as perceived by the partner in their interaction, can be affected by her/his gender (Slonim and Guillen, 2010), ethnicity or nationality (Glaeser et al., 2000; Fershtman and Gneezy, 2001), and multiple facial characteristics or expressions (Scharlemann et al., 2001; DeBruine, 2002; Campellone and Kring, 2012; Chen et al., 2012; Giang et al., 2012; Tortosa et al., 2013; Sofer et al., 2015). On the other hand, people often rely on prior social information, i.e., reputation, to infer the trustworthiness of the current partner (Delgado et al., 2005; Bracht and Feltovich, 2009; Chang et al., 2010; Charness et al., 2011; Fouragnan et al., 2013).

More often and more importantly, people assess trustworthiness by observing the behavior of a social partner in their interactions, especially in repeated interactions. This is an “interaction-based” learning process (Fouragnan et al., 2013) in which a trustor learn the trustee’s reputation through experience with her/him. To study this type of interactions, researchers in social decision making utilize a well-developed paradigm called the trust game that was first designed by Berg et al. (1995) (BDM trust game). The initial BDM trust game was a one-shot game between two anonymous persons. The trustor was first endowed with $10 and then decided how much to “invest” to the trustee. The amount invested was tripled and finally the trustee decided how much to pay back. Contrary to traditional economic theories, studies revealed that the trustor often invested and the trustee also paid back (Berg et al., 1995; Johnson and Mislin, 2011). It is suggested that this game measures trust and trustworthiness (Bracht and Feltovich, 2009). To address trust evolving and trustworthiness learning, studies have adopted the repeated version of the trust game that has a better ecological validity. Behavior studies, including those using mathematical models, have attempted to describe the reputation formation and learning dynamics during the repeated trust game (Anderhub et al., 2002; Cochard et al., 2004). These studies implicitly suggest that the strategies of both parties in the game follow the premise that the trustors make decisions based on the trustworthiness observed from the trustees’ behavior. There are also experiments manipulating the trustees’ trustworthiness and focusing on how the trustees’ behavior affected the trustors’ decisions in multi-round trust games (Chang et al., 2010; Campellone and Kring, 2012). They found that a trustor’s experience with the partner updated her/his belief of the partner and the subsequent decision. Moreover, this experience-based reputation overrode other social signals such as the partners’ facial trustworthiness or facial emotions.

Evaluation of trustworthiness with various kinds of information has also been investigated by neuroscience research. A number of studies have reported the neural representation of trustworthiness appraisals that were solely based on facial characteristics when previous social interactions were absent. While most of these studies used lesion and functional magnetic resonance imaging (fMRI) methods (Adolphs et al., 1998; Winston et al., 2002; Engell et al., 2007; Todorov et al., 2008; Castle et al., 2012; Mattavelli et al., 2012; Freeman et al., 2014), only a few event-related potential (ERP) studies have observed the electrophysiological correlates of trustworthiness evaluation when subjects saw different faces. Yang et al. (2011) explored subjects’ ERP time course during a simple evaluation task where they rated the facial trustworthiness of pre-categorized faces. The effects of facial trustworthiness on the earliest evoked visual component C1 (40–90 ms) and the late positive components (LPC, 400–600 ms) amplitudes were found in this study. Furthermore, only the LPC amplitude was found to be associated with subjective trustworthiness rating in the task. The authors attributed the C1 effect to the structural facial properties conveying cues about trustworthiness, while the trustworthiness effect on the LPC was interpreted as the attentional, affective or motivational aspects of facial trustworthiness processing. Another study also looked into the ERP differences between trustworthy and untrustworthy faces in a similar rating task (Marzi et al., 2014). The ERP components whose amplitudes varied with different subjective trustworthiness rating included the P100 (110–130 ms), an early posterior negativity (EPN, 200–350 ms) and the late positive potential (LPP, 300–500 ms). All of these components exhibited more pronounced amplitudes for subjectively rated untrustworthy compared to trustworthy faces. However, amplitudes of these components did not differ between different pre-experimental rated trustworthiness face categories. In another study where trustworthy or untrustworthy faces selected based on consensus judgments were paired with positive or negative personality traits, subjects’ ratings on the faces’ trustworthiness were affected by both perceptual and learned information (Rudoy and Paller, 2009). The ERP results suggested that perceptual information processing during trustworthiness appraisal was correlated with earlier (200–600 ms) ERPs in the anterior frontal sites while the effect of remembered information on this appraisal could be identified in a later (800–1000 ms) ERP correlate in the parietal sites. Although inconsistency remains among these three studies, it can be implied that during simple facial trustworthiness appraisal, earlier ERP components are associated with the rapid perception of certain physical facial characteristics embedding trustworthiness information. Later components, on the other hand, underlie more deliberate and emotional/motivational processing.

There is also literature regarding trustworthiness assessment and reputation learning during social interactions such as games and their neural bases. It has been demonstrated that people would depend more or less on the prior belief of the trustees to assess their trustworthiness, either in one shot trust games or during repeated investment. A couple of fMRI studies have identified the brain structures that encode the value of various reputation priors (Delgado et al., 2005; Stanley et al., 2012; Fouragnan et al., 2013) or the learned reputation and its effect on the trust behavior (Singer et al., 2004; King-Casas et al., 2005; Wardle et al., 2013). Among them, one study has shown that the activities of the caudate of the trustors’ brain differentiated between encountering good and bad trustees (Wardle et al., 2013). The authors put that this reflected the caudate’s role of maintaining information of outcomes and facilitating good decision making, as suggested in the reinforcement learning model, in a social decision making domain. Comparatively, less attention has been paid to the ERP mechanisms of reputation learning. The only two ERP studies, as far as we know, that aimed to uncover the reputation learning process in games were conducted by Osinsky et al. (2014) and Bell et al.’s (2016). In the research of Osinsky et al. (2014), a repeated ultimatum game, in which subjects interacted with fair or unfair proposers, was adopted. Subjects saw the face of a proposer each time before the monetary offer was presented. It is reported that only in the later period of the repeated interactions, could subjects differentiate reputation of the proposers. Furthermore, this differentiation was indicated by the discrepancy in the amplitude of the frontocentral cue-elicited feedback negativity (FN) when subjects saw the faces of proposers. This study suggested that learned reputation would be ascribed to the social partners after repeated interactions with them and the identity (i.e., face) of a partner would become a predictive cue for the fairness of the offer that followed. Moreover, the FN induced by the faces of the partners could be an indicator of learned reputation. Bell et al.’s (2016), on the other hand, adopted a prisoner’s dilemma game in their study and found an anterior positivity (400–600 ms) that was correlated with the retrieved reputation when a partner’s face was shown after several rounds of interactions. This ERP component differed only between the faces with established reputation and the control faces, but not between cooperator and cheater faces.

Despite some effort in related research fields, the evolution of trustworthiness appraisal in iterated trust games and its ERP correlates remain unclear. In this study, we aimed to investigate the trustors’ learning of their partners’ reputation from multi-round interactions by observing both behavioral performance and neural activities of them throughout this learning process. We adopted an ERP experiment in which subjects acted as trustors in a repeated trust game and play with several trustees alternately. There were both “good” and “bad” trustees who would generally or seldom reciprocate respectively. Subjects were not provided with the information of their partners’ trustworthiness throughout the game. Nonetheless, we predicted that subjects would get to know the trustees as their experience with each trustee accumulated. They would start with knowing little about their partners and end with recognizing the “good” and the “bad” to a large extent through learning. This interaction-based learning would be reflected in their investment decisions while the ascribed reputation to each trustee would finally be indexed by certain ERP components. Specifically, when the game was played repeatedly, subjects should become more likely to trust those partners who often reciprocated and avoid investing to those who were not. Besides, when the differentiation of trustworthiness evaluation was formed, it should also be reflected by the differentiation of the ERP time course related to trustworthiness appraisal.

Based on previous research, we were interested in several ERP components that may be involved in this study. First, we hypothesized that an anterior negative brain potential peaking ~250 ms (Nieuwenhuis et al., 2004b; Donkers et al., 2005; Hajcak et al., 2006; San Martín, 2012) after the face stimuli could be a candidate component, the amplitude of which would differ after subjects had learned the trustees’ reputation and their strategies had been guided by the trustworthiness evaluation. This ERP component, usually mentioned as the FN, has been shown to reflect a binary evaluation of outcomes (Nieuwenhuis et al., 2004a). A host of studies have demonstrated that the amplitude of this negativity is larger following negative compared to positive decision outcomes (Miltner et al., 1997; Gehring and Willoughby, 2002; Holroyd and Coles, 2002; Yeung and Sanfey, 2004; Sato et al., 2005; Hajcak et al., 2006; Santesso et al., 2012; von Borries et al., 2013; Meng and Ma, 2015). There are also studies suggesting that the association between the FN amplitude and feedback evaluation can be observed even in the absence of any executed actions before the feedback (Donkers et al., 2005; Yeung et al., 2005). Furthermore, some more recent studies (Walsh and Anderson, 2011; Osinsky et al., 2014) have extended the FN to an indicator of evaluation of the cue stimuli (coined as cue-elicited FN) when the valence of the outcome stimuli have transferred to the cues based on established rules or through evaluative learning. We supposed that when subjects had sufficiently formed differentiated evaluation of the two groups of trustees, the association between the faces of the trustees and the most probable monetary outcomes would be built. Thus, the valence of outcomes would transfer to the faces. As a result, subjects would form a rapid “good-vs-bad” evaluation seeing the faces when they have learned enough of the trustees’ reputation, which would be indexed by the cue-elicited FN. Specifically, an increased negativity of the FN should be elicited by the faces of untrustworthy partners when reputation was well learned in the late period of the repeated interactions.

Second, we also surmised that once subjects formed the impression of their partners’ trustworthiness in the late phase, the general emotional evaluation towards the faces should differ. Previous neuroscience research has posited that trustworthiness appraisals of faces involve an emotional face reaction in social settings (Winston et al., 2002; Singer et al., 2004; Engell et al., 2007; Yang et al., 2011; Stanley et al., 2012; Marzi et al., 2014). Thus, divergent neural responses toward the faces should also be reflected in magnitude differences of those late positive components including the P300 and LPP. These components were reported to be associated with emotional and motivational aspects of face processing (Langeslag et al., 2007; Grasso et al., 2009; Vico et al., 2010; Tortosa et al., 2013; Ma et al., 2015a) and have been found in previous studies of facial trustworthiness assessment (Yang et al., 2011; Marzi et al., 2014).

## Materials and Methods

### Participants

Twenty-two male students from Zhejiang University participated in this experiment. Two of the subjects were excluded from the final analysis due to excessive electroencephalography (EEG) recording artifacts. The remaining 20 subjects (mean age = 22.75 years, standard deviation (SD) = 1.74) were all right-handed, had normal or corrected-to-normal vision. They reported no history of psychiatric or neurological disorders. All subjects provided written informed consent before the experiment. All procedures involving the subjects were in accordance with the 1964 Helsinki Declaration and its later amendments or comparable ethical standards. The study was approved by the Institutional Review Board of Neuromanagement Lab, Zhejiang University, Hangzhou, China.

### Materials and Procedure

The basic experimental procedure required subjects to make a series of repeated investment decisions in a typical trust game setting. To better simulate the real-world situations where people usually have face-to-face interactions, we used eight male facial photographs to represent the trustees in the game (Chang et al., 2010). The photographs were selected from a dataset consisted of 24 young Chinese male facial photographs collected from the Internet. A calibration group of 177 males rated the attractiveness and the trustworthiness of these candidate faces on 7-point Likert scales (1 = extremely low on attractiveness/trustworthiness, 7 = extremely high on attractiveness/trustworthiness). The eight selected faces representing the trustees were close in both the attractiveness (mean = 2.889, *SE* = 0.177) and the trustworthiness (mean = 3.072, *SE* = 0.164) ratings. The photographs were all gray-scale, with the same clarity, luminance and size. The males on the photographs were full-face and in neutral facial expressions.

After arrival, subjects received a written instruction on the repeated trust game. They were told that in each round, they would decide whether to invest CNY ¥2 to the trustee; and if they did, the investment would quintuple and then the trustee might repay either CNY ¥5 or nothing. The basic paradigm is consistent with one of our own studies (Ma et al., 2015b). In the cover story, subjects were convinced that the eight trustees were students of Zhejiang University who had previously participated in similar trust games in our laboratory and we had collected their repayment decisions for this game. Therefore, subjects were informed of a nonreal-time interactive mode with real trustees but actually played against the computer. This manipulation adopted has been validated by several trust game experiments (Tzieropoulos et al., 2011; Tortosa et al., 2013; Wardle et al., 2013; Ma et al., 2015b). Among the eight pseudo-trustees, four were randomly assigned as trustworthy persons and would repay CNY ¥5 with a probability of 0.8 while the other four would “behave” untrustworthily, repaying CNY ¥5 only at a probability of 0.2 (Fouragnan et al., 2013). This assignment was reset when each subject started the task, so which four trustees were assigned to the trustworthy (untrustworthy) condition was different for each subject. Subjects were not explicitly told the number of the more or less trustworthy trustees or their repayment probabilities.

**Figure 1:**
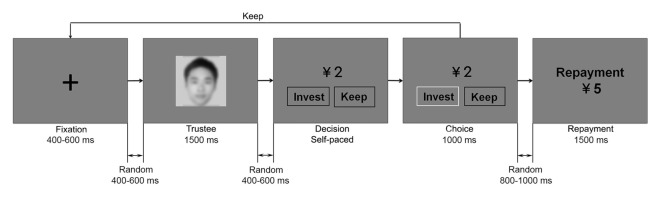
Experiment procedure. In each trial, the face of the trustee was shown first. Subjects had to choose between “invest” and “keep”. If they invested, the repayment of the trustee would then be revealed. If they kept the endowment, that round would end. In accordance with the research ethics of the journal, the face in this figure is obscured.

Subjects performed the experimental task comfortably seated 1 m away from the computer screen in an acoustically and electrically shielded room while their EEG was recorded. The task consisted of 240 trials, which were evenly divided into three blocks. These three blocks were designed to reflect a gradual process of learning, in which subjects could not have learned the trustees’ reputation at the very beginning and could have successfully recognized the “good” from the “bad” by the end of the game. Therefore, we focused on the first and the last blocks and the trials in the second block were considered to be similar to the filler trials in previous studies of social neuroscience (Wu et al., 2012; Qu et al., 2013; Osinsky et al., 2014; Ma et al., 2015c). For instance, in a recent study, to compare the behavioral and neural responses before and after successful learning, only data from the first (early) and the last (late) blocks was analyzed (Alperin et al., 2014). Presentation of stimuli on a 17″ CRT monitor and subjects’ keypad response recording were controlled by E-Prime software package (Psychology Software Tools, Pittsburgh, PA, USA). Each trial started with a fixation cross lasting for a random interval between 400 ms and 600 ms. After another random interval between 400 ms and 600 ms, the face of the trustee was presented for 1500 ms. Subjects would then see an endowment of CNY ¥2 and the two investment options (“invest” or “keep”) on the screen after a random interval between 400 ms and 600 ms. They needed to press the “1” or “3” key once they had made the decision. The positions of the two options and their corresponding key buttons were counterbalanced across subjects. The chosen option would then be highlighted by a color change of its frame for 1000 ms. Following a random interval between 800 ms and 1000 ms, the repayment of the trustee would be shown for 1500 ms. The inter-trial random interval was between 700 ms and 900 ms. Experimental paradigm is illustrated in Figure 1.

Upon the completion of all trials, subjects would leave the room and rate each trustee’s facial attractiveness and trustworthiness on a 7-point Likert scale (1 = “not attractive at all” or “not trustworthy at all”, 7 = “highly attractive” or “highly trustworthy”). After the rating, they pressed the “Enter” key on another computer to draw an integer that would decide which one of the 240 trials would count. Subjects would get a bonus of CNY ¥0, ¥2 or ¥5 according to the actual investment and repayment of that trial besides their show-up fee of CNY ¥40. Finally, subjects were informed of the pseudo-trustee manipulation, thanked and paid out.

### EEG Acquisition

During the task, EEG (band pass: 0.05–100 Hz; sampling rate: 1000 Hz) was recorded from 64 scalp sites according to the International 10–20 system with Ag/AgCl electrodes and a Neuroscan Synamp2 Amplifier (Scan 4.3.1, Neurosoft Labs Inc., Sterling, VA, USA). All electrodes were referenced to the left mastoid on-line and later off-line re-referenced to the linked mastoids. Vertical electrooculogram (EOG) was recorded with two electrodes placed above and beneath the left eye, while horizontal EOG was recorded with the other two placed at the outer canthus of each eye. The impedance was kept below 5 kΩ during recording.

### Data Analysis

For the behavioral performance, the percentages of the “invest” choice in both the trustworthy and untrustworthy trustee conditions in each block were calculated as investment rates. The investment rates were then submitted to a 2 (trustworthiness: trustworthy, untrustworthy) × 2 (phase: early, late) repeated-measures analysis of variance (ANOVA). The response times of the investment choices were analyzed using the same 2 × 2 repeated-measures ANOVA. The Greenhouse-Geisser correction was applied for the violation of the sphericity assumption in ANOVAs (uncorrected degrees of freedom are reported with corrected *p*-values), and multiple comparisons were corrected with the Bonferroni method when appropriate. Furthermore, the averaged post-experimental attractiveness and trustworthiness ratings to the two types of trustees also went into paired *t*-tests.

In the ERP data off-line analysis, the vertical ocular artifact correction used the regression approach described by Semlitsch et al. (1986). Digital filtering was applied using a 30 Hz low pass filter (24 dB/octave). Data in the time window between 200 ms before and 800 ms after the face stimuli presentation was segmented and baseline-corrected by the pre-stimuli period. Trials with baseline-to-peak deflections that exceeded ±80 μV were then excluded from averaging. For each subject, the averaged ERPs were then created for each electrode under both trustworthy and untrustworthy conditions in both early and late phases.

Based on previous research and visual inspection on the grand averaged ERP waveforms and the scalp distribution, we conducted statistical analyses on three ERP components. For the FN component (mean amplitude: 200–260 ms), data from F3, Fz, F4, FC3, FCz and FC4 electrodes were analyzed. For both the P3 (mean amplitude: 300–420 ms) and LPP (mean amplitude: 420–720 ms) components, data from CP3, CPz, CP4, P3, Pz, P4, PO3, POz and PO4 were analyzed. Amplitudes of these ERP components were submitted to repeated-measures ANOVAs to test the effects of three factors: trustworthiness (trustworthy, untrustworthy), phase (early, late) and electrode. The Greenhouse-Geisser correction was applied for the violation of the sphericity assumption in ANOVAs (uncorrected degrees of freedom are reported with corrected *p*-values), and multiple comparisons were corrected with the Bonferroni method when appropriate.

## Results

### Behavioral Results

Repeated-measures ANOVA showed that both trustworthiness (*F*_(1,19)_ = 94.718, *p* < 0.001) and phase (*F*_(1,19)_ = 12.062, *p* = 0.003) had significant effects on investment rate. Generally, subjects invested on trustworthy trustees (mean = 0.817, standard error (SE) = 0.029) more than untrustworthy ones (mean = 0.412, *SE* = 0.030) and their investment rate dropped through the early phase (mean = 0.659, *SE* = 0.024) to the late one (mean = 0.570, *SE* = 0.025). Furthermore, we found a significant interaction of trustworthiness and phase (*F*_(1,19)_ = 71.145, *p* < 0.001). Simple effect analysis firstly showed that investment rate was different in both phases. Subjects invested on trustworthy trustees (mean = 0.765, standard error (SE) = 0.030) more than untrustworthy ones (mean = 0.554, standard error (SE) = 0.034, *F*_(1,19)_ = 24.917, *p* < 0.001) in the early phase. In the late phase, the discrepancy in investment rate was more pronounced, with a rate of 0.869 (*SE* = 0.035) for trustworthy trustees and that of 0.271 (*SE* = 0.038) for untrustworthy ones (*F*_(1,19)_ = 131.727, *p* < 0.001). Furthermore, from the early to the late phase, investment rate for trustworthy trustees pronouncedly increased from 0.765 (*SE* = 0.030) to 0.869 (*SE* = 0.035; *F*_(1,19)_ = 12.477, *p* = 0.002) while this rate dramatically decreased from 0.554 (*SE* = 0.034) to 0.271 (*SE* = 0.038; *F*_(1,19)_ = 52.845, *p* < 0.001) for untrustworthy trustees.

The ANOVA on response time revealed no significant effect of trustworthiness (*F*_(1,19)_ = 1.764, *p* = 0.200) but a significant effect of phase (*F*_(1,19)_ = 25.220, *p* < 0.001). Subjects made faster decisions in the late phase (response time mean = 405.291 ms, *SE* = 40.157) than in the early phase (response time mean = 609.683 ms, *SE* = 53.115). No interaction of trustworthiness and phase was found (*F*_(1,19)_ = 0.383, *p* = 0.543).

Moreover, paired *t*-tests on the post-experimental ratings of the trustees’ trustworthiness and attractiveness showed that trustees in the assigned trustworthy group were perceived to be not only more trustworthy (trustworthy: trustworthiness mean = 5.438, *SE* = 0.204; untrustworthy: trustworthiness mean = 2.275, *SE* = 0.170; *t*_(19)_ = 11.392, *p* < 0.001) but also more attractive (trustworthy: attractiveness mean = 4.133, *SE* = 0.195; untrustworthy: attractiveness mean = 3.063, *SE* = 0.250; *t*_(19)_ = 4.477, *p* < 0.001) than those in the untrustworthy group.

### ERP Results

**Figure 2:**
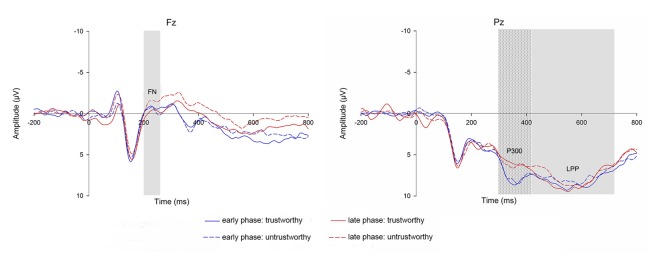
Grand averaged event-related potentials (ERPs) at Fz (feedback negativity, FN) and Pz (P300 and LPP) comparing the four conditions over trustworthiness (trustworthy vs. untrustworthy) and phase (early vs. late). Rectangular shadows indicate the time windows of each component.

**Figure 3:**
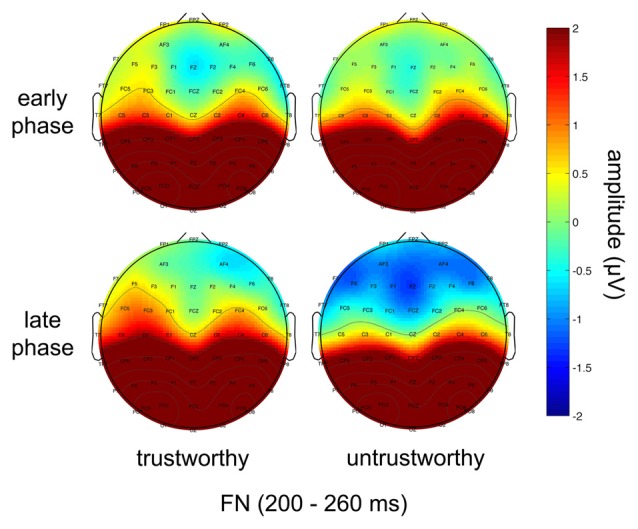
Topographical maps showing scalp distributions of the FN amplitudes in the trustworthy (left) and the untrustworthy (right) conditions in the early (upper) and the late (lower) phases.

The ERPs in the 2 (trustworthiness: trustworthy, untrustworthy) × 2 (phase: early, late) conditions are illustrated in Figure 2. Scalp topographies of the FN are shown in Figure 3.

#### FN

The ANOVA on the FN amplitude showed that neither the main effect of trustworthiness (*F*_(1,19)_ = 2.619, *p* = 0.122) nor that of phase (*F*_(1,19)_ = 0.173, *p* = 0.682) was significant, while a significant main effect of electrode was observed (*F*_(5,95)_ = 5.517, *p* = 0.004). FN amplitude reached negative maximum at Fz (mean = −0.620, *SE* = 0.701).

A significant interaction effect of trustworthiness and phase was manifested (*F*_(1,19)_ = 5.089, *p* = 0.036). An additional simple effect analysis revealed that in the early phase the FN amplitude difference was not significant in the two trustworthiness conditions (trustworthy: mean = 0.047, *SE* = 0.784; untrustworthy: mean = 0.076, *SE* = 0.721; *F*_(1,19)_ = 0.004, *p* = 0.948), but in the late phase the FN amplitude was significantly different in the two conditions (trustworthy: mean = 0.309, *SE* = 0.669; untrustworthy: mean = −0.703, *SE* = 0.761; *F*_(1,19)_ = 9.688, *p* = 0.006).

A significant interaction of trustworthiness and electrode was also found (trustworthiness × electrode: *F*_(5,95)_ = 3.474, *p* = 0.018). However, no other interaction effects were identified (phase × electrode: *F*_(5,95)_ = 0.393, *p* = 0.756; trustworthiness × phase × electrode: *F*_(5,95)_ = 0.904, *p* = 0.449).

#### P3 and LPP

The ANOVA on the P3 amplitude only revealed a significant effect of electrode (*F*_(8,152)_ = 12.856, *p* < 0.001), such that the P3 amplitude was largest at PO3 (mean = 9.171, *SE* = 1.055). However, no other effects were found (trustworthiness: *F*_(1,19)_ = 1.415, *p* = 0.249; phase: *F*_(1,19)_ = 2.789, *p* = 0.111; trustworthiness × phase: *F*_(1,19)_ = 0.844, *p* = 0.370; trustworthiness × electrode: *F*_(8,152)_ = 1.034, *p* = 0.385; phase × electrode: *F*_(8,152)_ = 1.539, *p* = 0.215; trustworthiness × phase × electrode: *F*_(8,152)_ = 0.539, *p* = 0.709).

Similarly, the ANOVA on the LPP amplitude found that none of the effects were significant (trustworthiness: *F*_(1,19)_ = 1.804 = 0.195; phase: *F*_(1,19)_ =, *p* = 0.931; electrode: *F*_(8,152)_ = 3.131, *p* = 0.057; trustworthiness × phase: *F*_(1,19)_ = 0.035, *p* = 0.854; trustworthiness × electrode: *F*_(8,152)_ = 1.261, *p* = 0.291; phase × electrode: *F*_(8,152)_ = 1.071, *p* = 0.364; trustworthiness × phase × electrode: *F*_(8,152)_ = 0.444, *p* = 0.796).

## Discussion

In the present study, we explored the learning process of evaluating others’ trustworthiness during repeated social interactions. In social interaction circumstances, trust is defined as the trustor’s willingness to accept vulnerability based on positive expectations of the actions of the trustee (Rousseau et al., 1998). Trustworthiness, then, is the reciprocity of the trustee that honors trust (Ashraf et al., 2006). Our study clearly shows that the partners’ trustworthiness strongly influences the trustors’ propensity to trust. The experiment data has proved that, overall, subjects invested more to those who often reciprocated. More importantly, subjects were getting better at evaluating their partners’ reputation as the interactions proceeded. Hence, they became more willing to invest in the trustworthy partners and drastically shrank from those who seldom repaid. It is worth noting that, although the investment rate has been different in the early phase, suggesting that there has already been some opportunity for reputation learning at that time, the discrepancy of investment rate significantly magnified in the late phase. This strategy adjustment reveals that they were learning to discriminate “good” trustees from “bad” ones throughout the game. Besides investment rate, the decreasing response time also suggests that subjects got more confident as they could gradually differentiate between two kinds of partners. Additionally, the result of the post-experimental trustworthiness rating is again a piece of evidence for this differentiation.

Subjects’ largely successful learning of their partners’ trustworthiness has been reflected by the electrophysiological dynamics during this repeated trust game. Our ERP results suggest that amplitude of the cue-elicited FN is associated with the differentiation of trustworthiness. In the early phase when subjects were not that clear about their partners’ characteristics, the FN amplitude was not significantly different when they saw the faces of the partners. In the late phase, however, a significant discrepancy of the FN amplitude was manifested, corresponding with the well-established investment discrimination between the two groups of partners. Hence, this component is proved to be a neural correlate of trustworthiness assessment in our experiment. The cue-elicited FN was more negative in the untrustworthy than the trustworthy trustee condition as hypothesized. This result is in line with the previous consensus that the FN, which maximizes at the medial frontal sites (Yeung et al., 2005; von Borries et al., 2013), is larger when an unfavorable stimulus is presented (Miltner et al., 1997; Gehring and Willoughby, 2002; Holroyd and Coles, 2002; Nieuwenhuis et al., 2004b; Yeung and Sanfey, 2004; Donkers et al., 2005; Sato et al., 2005; Yeung et al., 2005; Hajcak et al., 2006; Santesso et al., 2012; von Borries et al., 2013; Ma et al., 2015b).

Early research has shown that emotional contexts associated with faces could modulate very early (30–60 ms) sensory processing in visual areas and amygdala, which was reflected in magneto-encephalographic responses (Morel et al., 2012). In our experiment, the differentiation of the cue-elicited FN under different trustworthiness conditions in the late phase exhibits that trustworthiness appraisal can also be an immediate response when reputation was formed to a large extent, which is consistent with findings of existing behavior and ERP studies (Willis and Todorov, 2006; Todorov et al., 2009; Marzi et al., 2014). In these studies, evaluation on trustworthiness basing only on facial characteristics could be done within 100 ms and was reflected by early ERP components. Our result further shows that judgment of trustworthiness based on previous experience in social interactions can be formed in barely more than 200 ms, even when an explicit requirement of judging or decision making is absent. We again suggest that trustworthiness assessment should be a fast process that facilitates human social decision making.

Furthermore, trustworthiness appraisal embedded in the cue-elicited FN component evolved along with the learning of reputation when no prior information was provided. In our experiment, since each investment basically had a positive expected value (i.e., ¥0.5) when no information of the trustee’s reputation was available (i.e., the probability of getting repayment was equal to that of getting nothing), subjects were inclined to invest in each round at the very beginning. As the interactions advanced, they gradually recognized each partner’s reputation and adjusted their strategy. Therefore, when subjects saw the face of a trustee, they became more and more able to evaluate whether this man was likely to reciprocate their trust and whether they should invest in him. Unlike Bell et al.’s (2016) research where subjects also learned their partners’ reputation but the late anterior positivity only encoded whether there was retrieved socially salient memory (i.e., learned reputation) of a face, our study has shown an early ERP component that differed between two types of partners. When the reputation was sufficiently created, the faces of the trustees would become stimuli that afforded the “good-vs-bad” valence disparity derived from a learned association with the general investment outcomes of the trustees. As an electrophysiological indicator of the binary evaluation of the faces, the FN differed in amplitude in the late phase. Our results corroborate the reinforcement learning theory of the FN (Holroyd and Coles, 2002; Nieuwenhuis et al., 2004a; Hajcak et al., 2007) and existing studies that have found the valence-based amplitude disparity of the cue-elicited FN (Dunning and Hajcak, 2007; Baker and Holroyd, 2009; Liao et al., 2011; Walsh and Anderson, 2011). The modulation on cue-elicited FN amplitude by the learned reputation in the late phase is also consistent with the results found by the previously mentioned ERP study of reputation learning during a repeated ultimatum game (Osinsky et al., 2014). However, unlike the certain or probabilistic simple cues that predicted the favorableness of following outcomes based on fixed rules or the social cues that were completely indicative of the upcoming payoff after learning in social interactions, the faces in our experiment were less predictive but more instructive to what should happen next. Therefore, the face-induced FN in this study reflected not only the reputation assessment but also an instruction to the later investment decision. Our study complements findings of Osinsky et al. (2014) by providing similar neural evidence in a different repeated trust game, which also suggests that the cue-elicited FN may be a neural index of reputation learning in repeated social interactions. Besides, our ERP results are in line with previous fMRI research regarding reputation learning in trust games (Wardle et al., 2013), suggesting that when the identities of the trustees act as cues, they maintain information that guides decision making and supporting a reinforcement learning model during the trustors’ learning process.

In the late components, however, we did not find any differentiation corresponding with the learning process through the early to the late phase of the game. The posterior P300 and LPP amplitudes did not differ in the two trustworthiness conditions when subjects had already learned most of their partners’ reputation, which is contrary to the results of some studies regarding trustworthiness appraisals of faces (Yang et al., 2011; Marzi et al., 2014). One possible reason for this inconsistency is the difference in experiment design of our study and the others. Our experiment did not ask subjects to explicitly rate the trustworthiness of the trustees. Besides, subjects’ implicit evaluation on the trustworthiness of the trustees was based on social experience. In the studies of Yang et al. (2011) and Marzi et al. (2014), participants resorted to those common physical characteristics to infer trustworthiness instead. Actually, in a previous research regarding face evaluation, similar findings suggesting that the direction of the relationship between stimuli valence and amplitudes of the late positive components was inconsistent were also reported. The authors suggested that this seemingly contradictory finding might be the result of the discrepancy in experimental paradigm (Chen et al., 2012). On the other hand, based on the assumption that trustworthiness appraisal is a generalization of emotion evaluation, the studies on facial trustworthiness and the late components attributed the difference of P300 or LPP to the motivational difference of emotion induced by different faces. However, fMRI studies regarding the relationship between amygdala activation and trustworthiness evaluation, which also posited that the amygdala processed the emotional stimuli, have demonstrated inconsistent results on the direction of this relationship (Adolphs et al., 1998; Winston et al., 2002; Singer et al., 2004; Engell et al., 2007; Todorov et al., 2008; Mattavelli et al., 2012; Freeman et al., 2014). In addition, a relatively small sample size may not fully reveal a potential learned trustworthiness effect on P300/LPP in our study. Therefore, the neural response underlying the emotional processing of learned facial trustworthiness needs further investigation in future research, especially that with large sample sizes, which is beyond the scope of the present study.

Interestingly, we have found that the post-experimental facial attractiveness rating was significantly different between the two trustworthiness conditions. Subjects rated faces of those more trustworthy trustees as more attractive. We think that this discrepancy of rating cannot be accounted by facial attractiveness differences of the face stimuli. First, before the experiment, the faces were similarly rated in attractiveness by the calibration group. Second, each face was randomly reassigned to one of the trustworthiness conditions so that the same face was not always placed in the same condition. These manipulations should have excluded facial attractiveness from the factors that influenced the behavioral or ERP results (see Chen et al., 2012). We assume that our result manifests “what is good is beautiful”, which has been suggested by existing research on facial attractiveness judgment showing that positive personality traits could enhance a person’s facial attractiveness rated by others (Zhang et al., 2014). Besides, a neuroscientific study has revealed that activation of some brain regions increase/decrease as a function of both attractiveness and goodness, providing some implications for understanding why judgments of these two dimensions are usually highly correlated (Tsukiura and Cabeza, 2011). Our study, however, involved a learned trustworthiness evaluation and showed its contribution to facial attractiveness rating. Thus, it implies that repeated interactions can not only form our judgment to others’ social reputation but also influence our perception of their physical features.

## Conclusion

The present study investigated a process in which the trustors learned the trustees’ trustworthiness by observing their behavior and adjusted their own trust decisions accordingly. The ERP results revealed that magnitudes of the cue-elicited FN varied as whether the trustors saw the trustworthy or untrustworthy trustees, but only in the later period of the repeated trust game. Therefore, we suggest the cue-elicited FN as an early ERP index of reputation appraisal in repeated social exchanges, which corroborates and complements previous findings (Osinsky et al., 2014). In summary, our study demonstrates that one’s implicit rating of social partners’ trustworthiness that is gradually formed through interactions with them will affect her/his trust behavior and the gradual differentiation of the cue-elicited FN component reflects this learning process.

## Author Contributions

DL and LM conceived the study and designed the experiment. DL ran the experiment, analyzed the data and drafted the manuscript. DL, LM and QM revisited the manuscript.

## Conflict of Interest Statement

The authors declare that the research was conducted in the absence of any commercial or financial relationships that could be construed as a potential conflict of interest.
