# Corrigendum: Who Deserves My Trust? Cue-Elicited Feedback Negativity Tracks Reputation Learning in Repeated Social Interactions

**DOI:** 10.3389/fnhum.2019.00083

**Published:** 2019-03-06

**Authors:** Diandian Li, Liang Meng, Qingguo Ma

**Affiliations:** ^1^School of Management, Zhejiang University, Hangzhou, China; ^2^Beijing Xinsight Technology Co. Ltd., Beijing, China; ^3^Neuromanagement Lab, Zhejiang University, Hangzhou, China; ^4^School of Business and Management, Shanghai International Studies University, Shanghai, China; ^5^Laboratory of Applied Brain and Cognitive Sciences, Shanghai International Studies University, Shanghai, China; ^6^Institute of Neural Management Sciences, Zhejiang University of Technology, Hangzhou, China

**Keywords:** trustworthiness, trust game, social learning, event-related potential, feedback negativity

There was an error in the **Funding** statement. The correct number for the National Project is “AWS14J011.”

The authors apologize for this error and state that this does not change the scientific conclusions of the article in any way. The original article has been updated.

